# Thousand-Fold Enhancement of Photothermal Signals in Near-Critical CO_2_

**DOI:** 10.1021/acs.jpcc.2c08575

**Published:** 2023-02-09

**Authors:** Yonghui Wang, Subhasis Adhikari, Harmen van der Meer, Junyan Liu, Michel Orrit

**Affiliations:** † Huygens-Kamerlingh Onnes Laboratory, Leiden University; 2300 RA Leiden, The Netherlands; ‡ School of Mechatronics Engineering, Harbin Institute of Technology; Harbin 150001, P. R. China

## Abstract

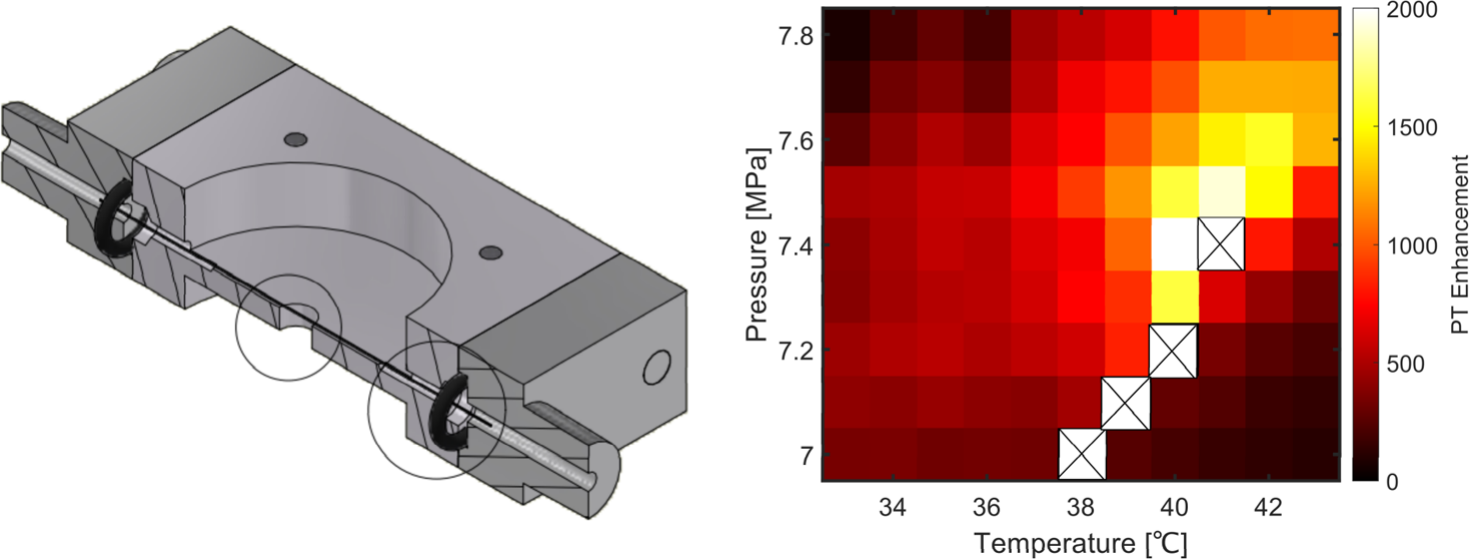

Photothermal (PT) microscopy has shown strong promise in imaging single absorbing nano-objects in soft matter and biological systems. PT imaging at ambient conditions usually requires a high laser power for a sensitive detection, which prevents application to light-sensitive nanoparticles. In a previous study of single gold nanoparticles, we showed that the photothermal signal can be enhanced more than 1000-fold in near-critical xenon compared to that in glycerol, a typical medium for PT detection. In this report, we show that carbon dioxide (CO_2_), a much cheaper gas than xenon, can enhance PT signals in a similar way. We confine near-critical CO_2_ in a thin capillary which easily withstands the high near-critical pressure (around 74 bar) and facilitates sample preparation. We also demonstrate enhancement of the magnetic circular dichroism signal of single magnetite nanoparticle clusters in supercritical CO_2_. We have performed COMSOL simulations to support and explain our experimental findings.

## Introduction

Single-particle and -molecule spectroscopy^1,2^ has become a standard tool for nanoscale imaging in biological systems and nanomaterials. Although most single-molecule studies are so far based on fluorescence, in the last few decades several fluorescence-free approaches^3,4^ have been developed. Among them, photothermal (PT) microscopy^5−11^ has shown strong promise. The sensitivity of PT microscopy enables the detection of the absorption of a single 1 nm gold nanoparticle (AuNP)^12^ and of single non-fluorescent molecules^13^ at room temperature. However, these highly sensitive PT measurements required high laser powers, which preclude application to systems which cannot sustain such high excitation powers. For example, imaging single conjugated polymers^14−16^ or perovskite nanocrystals^17^ requires low excitation powers, typically well below 1 μW in a diffraction-limited focal area. Single quantum dots have been imaged by PT contrast earlier,^18^ but these measurements required laser powers at which quantum dots became non-fluorescent. Simultaneous absorption and fluorescence imaging of single quantum dots, which has the potential to provide detailed insight into the mechanism of photoblinking, would require much lower excitation power. Imaging single metallic nanorods^19^ and other non-spherical nanoparticles, which are prone to reshaping at moderate temperatures, also requires low heating laser powers.

Most PT experiments use a heating laser (power *P*_heat_) and a probe laser (power *P*_probe_). Therefore, the signal (*S*) is proportional to both powers and to the thermo-refractive coefficient (d*n*/d*T*) of the imaging medium as
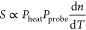


Organic liquids such as glycerol and pentane are typically used as PT media for imaging single nano-objects. Their thermo-refractive factors, d*n*/d*T*, are 2.77 × 10^–4^ K^–1^ for glycerol and 5.99 × 10^–4^ K^–1^ for pentane. Because of such a low d*n*/d*T* value, high heating and probe laser powers (typically in the order of tens of mW in a diffraction-limited area) are required to achieve a sensitive PT detection of single weakly absorbing nanoobjects. Chang and Link^6^ showed that using a thermotropic liquid crystal such as 5CB as a medium, PT signals can be enhanced 20 times compared to glycerol due to the high d*n*/d*T* value of 5CB. Parra-Vasquez et al.^20^ showed that near the nematic-to-isotropic phase transition of a liquid crystal, the PT signal can be enhanced 40 times compared to water as a PT medium. However, such phase transitions require long equilibration times. For a gas near its critical point, d*n*/d*T* increases steeply. For example, in xenon as shown in Figure S3, the d*n*/d*T* value can exceed 10^–2^ K^–1^ near the critical point, a value 100–1000 times larger than those of standard organic liquids. Thermal relaxation times in near-critical xenon, however, are much shorter than in mesophases such as liquid crystals because the simple atomic fluid does not require the slow molecular motions at work in complex molecular liquid crystals. Because of the high d*n*/d*T* value and the short relaxation time, critical simple liquids are better enhancers of the PT signal than liquid crystals. In our previous study,^21^ we have shown that the PT signal can be enhanced more than a thousand times in near-critical xenon compared to that in glycerol. It is worth mentioning here that even higher enhancements of the PT signal are possible when the system is placed close to the liquid–gas phase transition.^22^ However, such processes are highly nonlinear and hard to control to obtain stable signals. Working very close to the critical point requires a high degree of temperature control. Even minute temperature fluctuations result in large fluctuations of the PT signal. In particular, large absorbing nanoobjects or high laser powers lead to heating that removes the surrounding liquid from its critical point. Therefore, this method is best applied to weakly absorbing nano-objects and/or to low excitation powers. The method cannot be used to improve the signal-to-noise ratio of photostable samples because the temperature range is limited to the near-critical region. Rather, it enables studies of weakly absorbing and photosensitive samples.

Supercritical pressures are relatively high. The critical pressure of xenon is 58 bar and that of CO_2_ is 73 bar. Our previous imaging^21^ in near-critical xenon required a tedious sample preparation and a complex high-pressure cell. This cell was limited to pressures up to 65 bar because of glass fracture at high pressure. A further disadvantage was the high price of xenon. The inert gas CO_2_ is much cheaper than xenon, while its critical temperature (*T*_c_ = 31 °C) is slightly higher than room temperature. For xenon, the critical temperature *T*_c_ = 16 °C requires cooling from room temperature. As the critical pressure (*P*_c_) of CO_2_ is slightly higher than that of xenon, we had to re-design our previous cell.^21^ In this study, we report a capillary-based design working at pressures up to hundreds of bars, which is well suited to supercritical CO_2_. We demonstrate about 2000-fold enhancement of the PT signal in supercritical CO_2_ for single 30 nm AuNPs.

Photothermal circular dichroism (PT CD) microscopy has recently been demonstrated for the detection of CD in single nanoparticles.^23,24^ CD is the differential absorption of left- and right-circularly polarized light. PT CD microscopy also enables imaging the magnetization of single magnetic nanoparticles, i.e., photothermal magnetic CD (PT MCD) microscopy.^25^ The detection sensitivity of PT MCD can be enhanced using a supercritical liquid as a PT medium. Herein, we demonstrate enhancement of the MCD signal of single magnetite nanoparticle clusters in supercritical CO_2_.

## Experimental Section

### Design of the Pressure Cell

**Figure 1:**
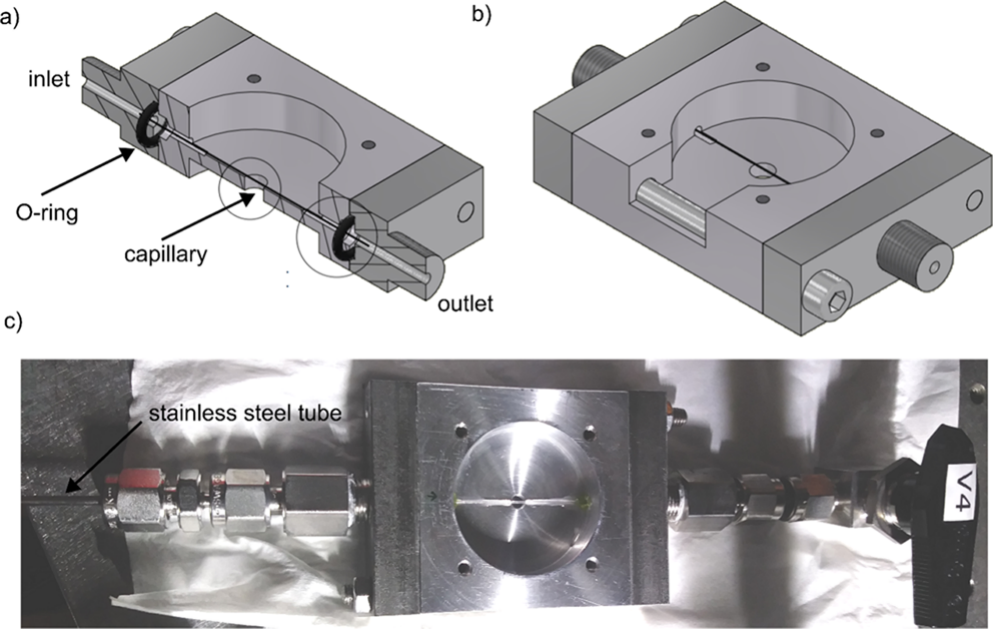
(a,b) Schematic representation of the pressure cell. The pressure cell has an inlet and an outlet. The inlet is connected to a high-pressure gas cylinder with a stainless steel tube. The inlet and outlet parts are connected to the pressure cell by O-rings. The pressure cell has a small hole in the center to pass the transmitted light, where the objective focuses the light onto the sample. (c) Picture of our pressure cell containing a capillary sample.

Figure 1a,b shows a schematic design of our capillary sample holder, called here “pressure-cell”. A picture of the sample holder is shown in Figure 1c. The pressure cell contains an inlet to purge CO_2_ inside the capillary and an outlet to purge out CO_2_ from the capillary. The square capillary (CM Scientific 8330-50) is glued to the pressure cell holder with epoxy glue. A stainless steel tube is used to flow CO_2_ from a high-pressure gas cylinder to the pressure cell. At first, low-pressure CO_2_ gas is passed through the capillary for tens of seconds to remove the air inside the tube and capillary. Thereafter, the outlet is closed, and the pressure is slowly increased using the piston-based method described in ref (21).

Our new capillary-based pressure cell allows us to work at pressures higher than 500 bar. The capillary’s inner diameter is 300 μm, and its thickness is 150 μm. The maximum pressure before breaking such a capillary would be several hundreds of bars,^26,27^ which is sufficient to work with supercritical CO_2_.

### Sample Preparation

The glass square capillary is first cleaned with piranha solution which also makes the capillary hydrophilic. Then the capillary is chemically functionalized with an APTES ((aminopropyl)triethoxysilane) solution (1:10 dilution of APTES in water) to bind gold particles to the glass surface directly from the solution, instead of spin-coating them. An aqueous suspension of metallic nanoparticles or magnetite nanoparticles is filled inside the capillary using capillary action. The solution is kept inside the capillary for about 5 min to bind the nanoparticles on the capillary surface. After that, the solution is flushed with distilled water and dried with dry N_2_ gas.

### Optical Setup

The details of the optical setup are described in ref (25). A heating laser of wavelength 532 nm is passed through a combination of polarization modulators (electro-optic modulator and photo-elastic modulator) to modulate the light between left and right circularly polarizations. A linear polarizer is inserted for intensity modulation during the PT measurement. After the polarizer, a lens is used to focus the light into the back-focal plane of the objective so as to achieve Koehler illumination. The details about the Koehler illumination in PT CD can be found in ref (24). For the standard confocal PT measurement, we take out the lens to focus the collimated beam at the capillary with a high-NA objective. To probe the thermal lens created by the heat released from a nanoparticle upon absorption of the heating beam, a collimated circularly polarized probe beam at 780 nm is focused at the capillary with the high-NA objective through standard immersion oil (Olympus, IMMOIL-F30CC). The probe beam scattered by the thermal lens is afterward collected by the same objective. The modulation of the scattered probe signal at the modulation frequency of the heating beam is detected as a PT signal using a sensitive lock-in amplifier.

### Simulation

We used a COMSOL model to calculate the absorbed power by a nanoparticle illuminated with a circularly polarized plane wave. Details about the COMSOL model can be found in ref (28). The absorbed power creates a temperature profile *T(r)* surrounding the nanoparticle and consequently creates a refractive index profile *n(r)*. Index modulation by the modulated heating beam scatters the probe beam and produces an interference signal *E*_ref_*× E*_sc_*. E*_ref_ and *E*_sc_ are the electric fields for the reference and scattered beams, respectively. In our case, the reference beam is the reflection at the glass–medium (either hexadecane or CO_2_) interface. Note that we consider here the steady-state approximation. The PT signal is calculated as the difference in the integrated interference signal with the heating laser on and off as
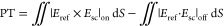
where “on” and “off” subscripts are for the heating laser on and off, respectively. The integral runs over a surface surrounding the particle, taken only for the backward detection as our PT microscope is reflection-based.

Using the COMSOL simulation, we have calculated the PT signal of a single 30 nm AuNP in both hexadecane and CO_2_. For CO_2_, we have calculated PT signals at different temperatures and pressures. Subsequently, we have calculated the enhancement factor for the PT signal in CO_2_ with respect to that in hexadecane. Figure 3b shows the enhancement factor map at different temperatures and pressures near the critical point of CO_2_. The simulation shows that near the critical point of CO_2_ (i.e., at 31 °C and 7.37 MPa), the enhancement factor is more than 1000 with a maximum enhancement factor of 1500 at the critical point. We have also performed the same simulation for xenon. The enhancement factor map in xenon is shown in Figure S1. The maximum enhancement factor obtained for xenon (i.e., 4200) is about a factor of 2.8 times higher than that in CO_2_. Note that below the critical point (*T*_c_, *P*_c_) of CO_2_, near three data points (i.e., 31 °C, 7.2 MPa; 30 °C, 7.1 MPa; and 29 °C, 7.0 MPa) the PT signal varies discontinuously due to the liquid–gas phase transition^22^ (Figure 3b). We have obtained a similar behavior for xenon (Figure S1).

The so-called PT figure of merit, i.e., (*1*/*k* × *n* × d*n*/d*T*, where *k* is the thermal conductivity, *n* is the refractive index, and d*n*/d*T* is the thermo-refractive coefficient) as defined in ref (29), provides a qualitative estimate of the PT signal. We have calculated the enhancement factor map for the figure of merit in CO_2_ and xenon as shown in Figures S2 and S3, respectively. The maximum enhancement factors obtained for CO_2_ and xenon are about 125 and 316, respectively. The significant difference between the maximum enhancement factor obtained from the COMSOL simulation and that based on the figure of merit is due to the more accurate consideration of the reflection at the glass–medium interface in the simulation, which is obviously not included in the figure of merit calculation. The reflection at the glass–medium interface in CO_2_ and xenon is different from that in hexadecane and varies near the critical point depending on the temperature and pressure.

Next, we performed the same COMSOL simulation for the CD signal. The CD signal is calculated as the differential PT signal between left- and right-circularly polarized light. To calculate the CD signal, we considered a nanoparticle with a g_CD_ factor of 0.02. The g_CD_ factor is the CD signal normalized by the PT signal. Similar to the PT enhancement factor map, an enhancement factor map of the CD signal is also calculated for CO_2_ as shown in Figure S4. The CD enhancement factor map looks similar to the PT enhancement factor map. We have calculated the g_CD_ factor from the simulation by normalizing the CD signal with the PT signal, and we plotted the g_CD_ factor map in Figure S4. We can see that the g_CD_ factor obtained from the simulation is quite close to the g_CD_ factor of 0.02 which is set in the simulation, with a deviation mostly below 10%.

## Results and Discussion

We first checked the image quality of single-particle imaging inside a square capillary. We measured the three-dimensional point-spread function (PSF) of the PT signal of a single magnetite nanoparticle in hexadecane (Figures S5 and S6). The lateral and longitudinal PSF sizes are 0.75 and 3.1 μm, respectively. The PSF sizes are significantly larger than the diffraction-limited sizes obtained in standard confocal microscopy. In the present case, because the heating beam is weakly focused, the PT PSF is close to that of the probe beam. The capillary reduces the effective numerical aperture and thereby the spatial resolution (see Supporting Information). We have also measured the PSF of the MCD signal of the same magnetite particle. The MCD PSF is shown in Figures S5 and S6 and looks similar to the PT one, indicating that there is no additional imaging artifact in the CD mode. The probable reasons for the larger PSFs are as follows. First, the thickness of the capillary is 150 μm, whereas the objective is corrected for a thickness of 170 μm. The mismatch in thickness may create spherical aberration. Second, there is a slight mismatch in the refractive index of hexadecane (1.43) and immersion oil (1.51). Third, because of the small capillary size (0.6 mm), the illumination angle of the high-NA objective is reduced, which effectively lowers the NA of the illumination (the illumination area is about 1.12 mm which is much larger than the capillary width of 0.6 mm). The reduction in NA due to lost rays at the upper capillary surface leads to a loss of resolution and to a broadened PSF. It is worth mentioning here that a round capillary would induce more aberrations because of its non-flat and anisotropic surface.

**Figure 2:**
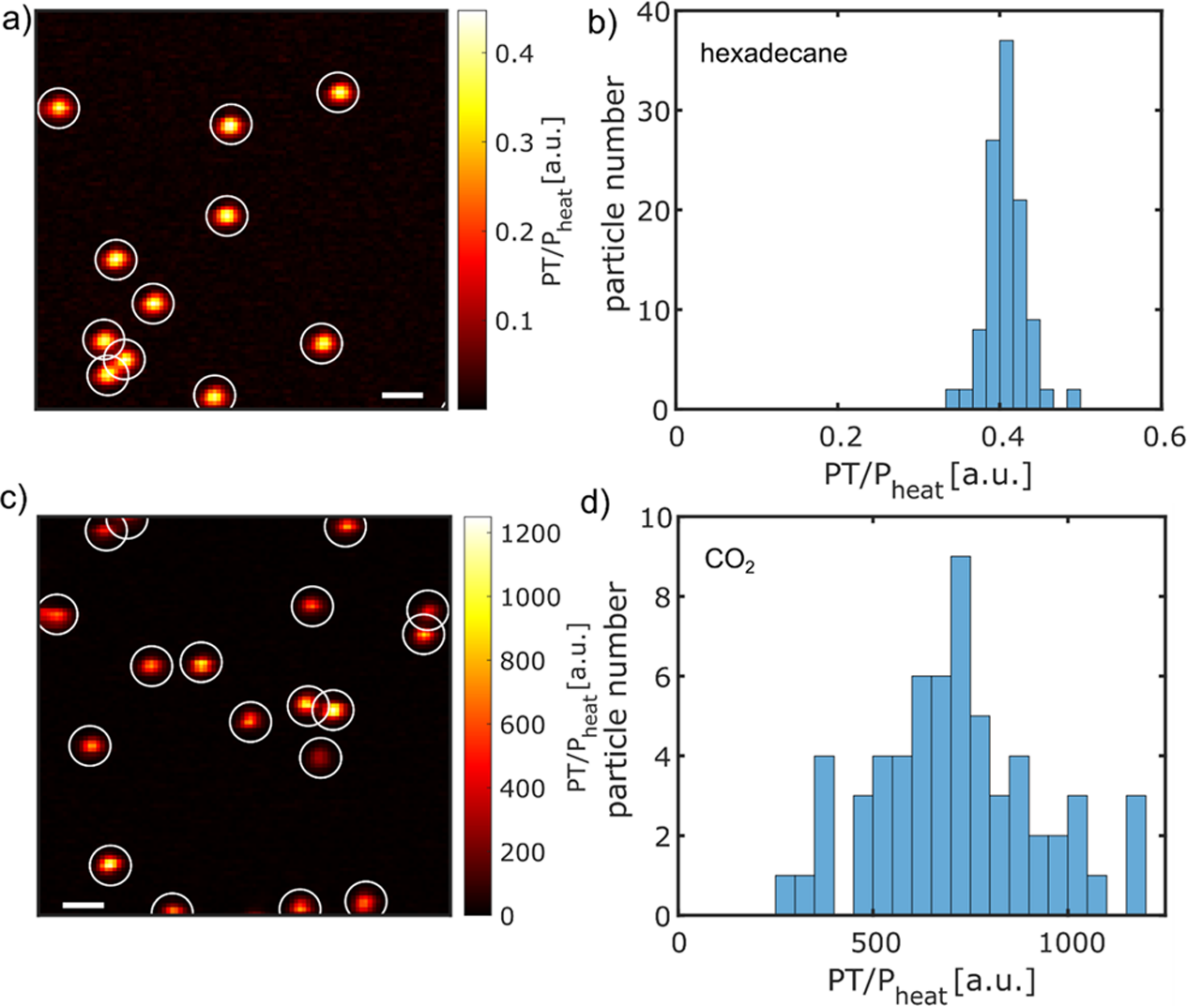
(Left) PT image and (right) histogram of PT signals in (top) hexadecane and (bottom) CO_2_ at the critical point, 40 °C and 7.4 MPa. The heating and probe laser powers were 3 mW and 70 μW, respectively, in hexadecane and 10 μW and 70 μW, respectively, in CO_2_. Scale bar = 1 μm. The probe laser power is the same in both cases; however, the heating laser power is different. Therefore, we normalized the PT signals with the heating laser power to compare the two cases. Comparing the two histograms, one can see that the signal is enhanced about 1800 times in CO_2_ compared to that in hexadecane.

As a next step, we measured PT signals of single 30 nm AuNPs in hexadecane to compare with PT signals in supercritical CO_2_. For these measurements, the heating and probe beams were focused on the sample as in standard confocal PT microscopy. Figure 2a shows PT images of several single AuNPs in hexadecane. All the particles show homogeneous PT signals. Figure 2b shows a histogram of PT signals of 110 single AuNPs. The histogram shows a narrow distribution indicating that all the particles are single particles. The heating and probe laser powers were 3 mW and 70 μW, respectively, in a diffraction-limited focused spot.

**Figure 3:**
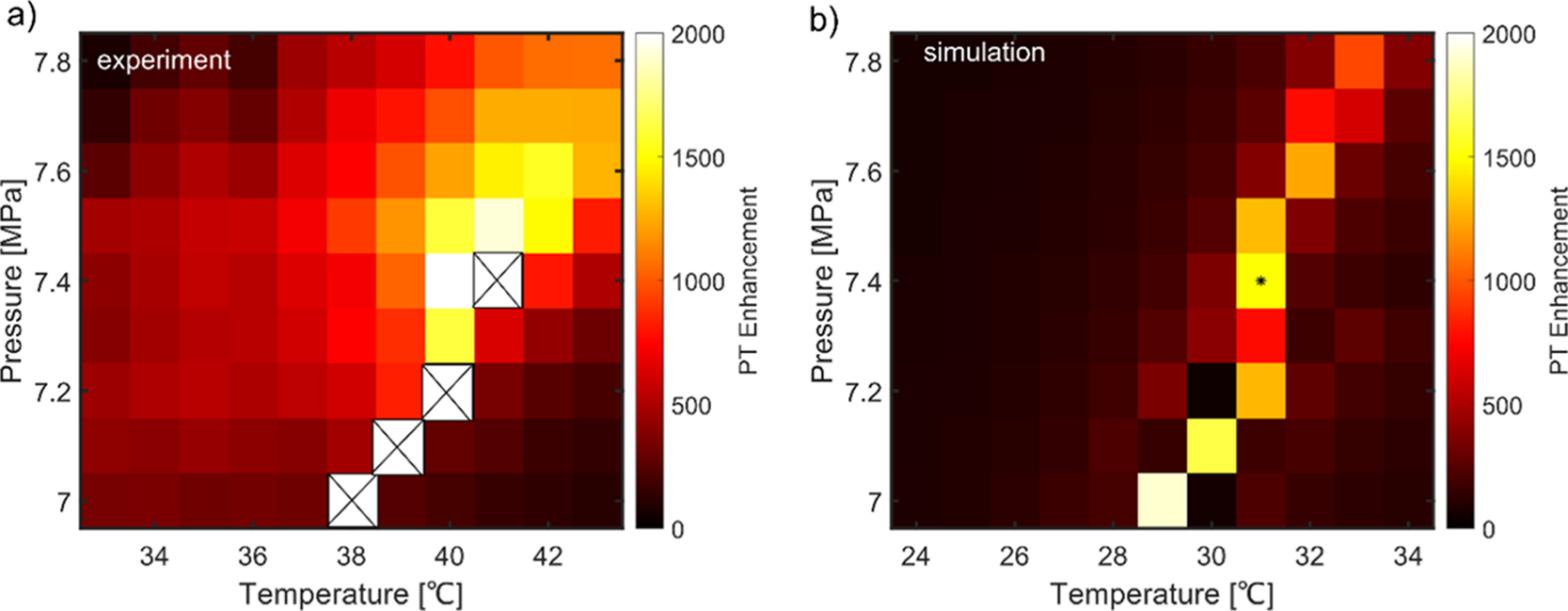
PT enhancement factor map measured in CO_2_ in the temperature range of 31 to 43 °C and in the pressure range of 7 to 7.8 MPa. The maximum enhancement is obtained at 40 °C and 7.4 MPa. At a few temperatures and pressures marked by cross symbols, the PT signal showed large fluctuations; these points are excluded from the map. (b) PT enhancement factor map at different temperatures and pressures calculated using the COMSOL simulation.

To measure the PT signals near the critical point of CO_2_, we varied the temperature and pressure. At each temperature and pressure, an average PT signal is calculated from the PT signals of 12 single AuNPs. The enhancement factor is calculated as the mean PT signal in CO_2_ normalized by the mean PT signal in hexadecane. A map of the PT enhancement factor as a function of temperature and pressure is shown in Figure 3a. The maximum enhancement factor obtained from the experiment matches well with the prediction from the simulation as shown in Figure 3b. However, the maximum enhancement in the simulation obtained is at the critical point, i.e., 31 °C and 7.4 MPa, whereas the maximum enhancement obtained in the experiment is at 40 °C and 7.4 MPa. We assign the difference in temperature of 9 K to the temperature difference between the probe (in contact with the sample holder) and the capillary (Figure S8), which is cooled down by heat diffusion to the objective at room temperature (20 °C). The objective at room temperature acts as a heat sink. Nevertheless, a maximum enhancement factor of about 2000 is obtained at 40 °C and 7.4 MPa.

To compare the PT signals in CO_2_ with those in xenon, we have also measured PT images of several single 30 nm AuNPs in xenon inside a capillary near the critical point. The PT enhancement factor map of xenon is shown in Figure S7. The maximum enhancement factor obtained for xenon is about 2200, which is similar to that in CO_2_. From the simulation, we found that the maximum enhancement factor in xenon is about 4200 (see Figure S1). The mismatch between the experiment and simulation is probably due to a low purity of the xenon gas. CO_2_ being much cheaper can be flushed more often than xenon. Note that in the case of xenon, we obtain the maximum enhancement factor at 16 °C and 5.9 MPa which is similar to the prediction from the simulation. The temperature gradient with the objective is, however, 3–4 times lower in absolute value than with CO_2_.

To have better statistics, we measured the PT signals of 61 single 30 nm AuNPs in CO_2_ at the critical point, 40 °C and 7.4 MPa. An example of a PT image is shown in Figure 2c. As compared to that of hexadecane, the PT signals of single particles look more heterogeneous as confirmed by the distribution of PT signals (Figure 2d). The distribution in CO_2_ is much broader than that in hexadecane. We assign this large heterogeneity in signal to the proximity of the critical point and to the large influence of small temperature variations on the enhancement and thereby on the strength of the signal. Slight variations in particle size or in the particle’s contact area with the glass surface can cause variation in heat conduction and in particle temperature, thereby broadening the distribution of PT signals. The contact area of a nanoparticle with a mirror depends on crystal facets and can differ strongly from particle to particle.^30^ Therefore, for quantitative measurements, it is probably better to measure PT signals near the critical point but not too close to it. Here, we measured at the critical point to demonstrate experimentally the maximum enhancement of PT signals. We also noticed that at a certain temperature and pressure as marked by the cross sign in Figure 3a, PT signals show large temporal fluctuations over time. The large fluctuations at the temperatures and pressures below the critical point are due to the liquid–gas phase transition at which nanobubble formation occurs.^22^ The large fluctuation at 41 °C, 7.4 MPa is not well understood. We speculate that due to high d*n*/d*T* values at this measurement point, any small perturbation would cause a large change in the PT signal. Nevertheless, one needs to be careful in interpreting the spatial inhomogeneity or temporal fluctuations of the PT signal near the critical point. Note that in earlier experiments in near-critical xenon,^14^ the particles were spin-coated on a glass slide with a polymer layer, which ensured good thermal contact with the slide. Here, we could not spin-coat the particles inside the capillary, and their contact with the surface was less good.

**Figure 4:**
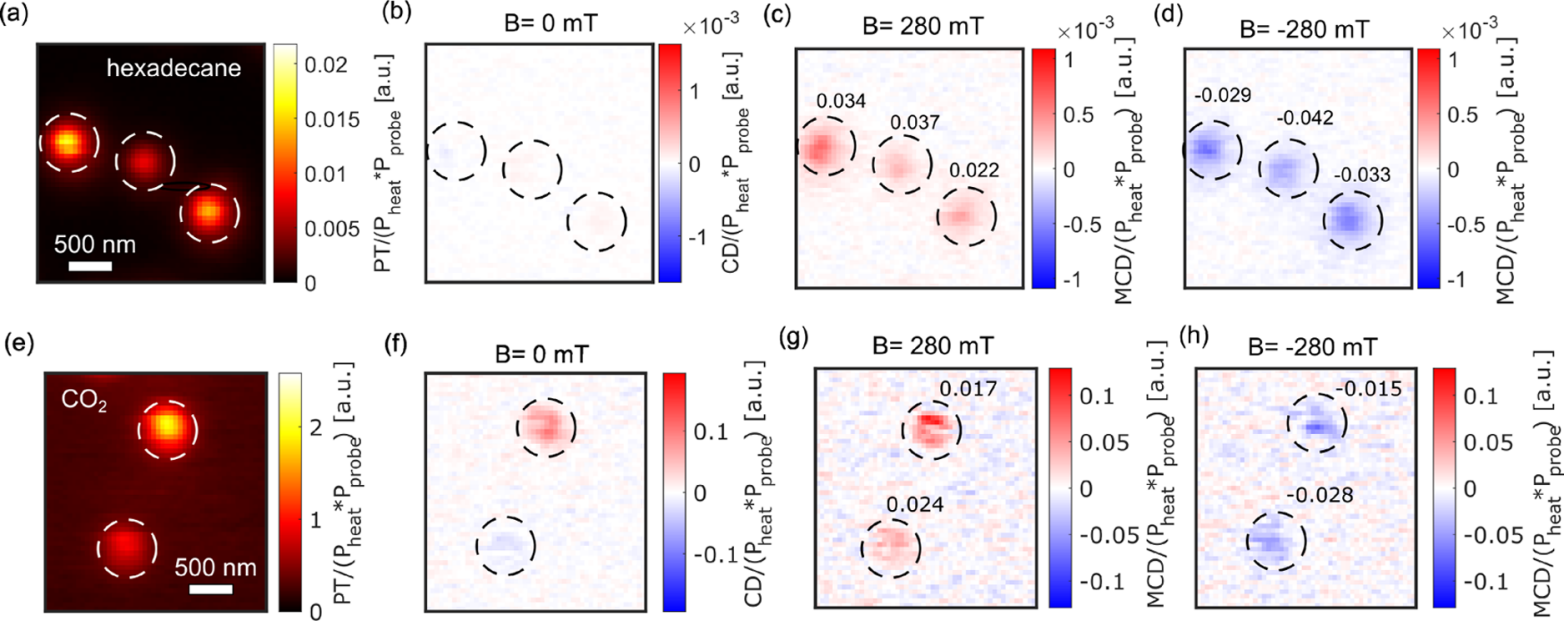
(a) PT, (b) CD at *B* = 0 mT, (c) MCD at *B* = + 280 mT, and (d) MCD at *B* = – 280 mT of three single magnetite nanoparticle clusters in hexadecane. The heating and probe powers were 102 mW and 18 mW, respectively. (e) PT, (f) CD at *B* = 0 mT, (g) MCD at *B* = + 280 mT, and (h) MCD at *B* = – 280 mT of two single magnetite nanoparticle clusters in CO_2_. The heating and probe powers were 15.5 and 1 mW, respectively. As the heating and probe laser powers are different in two cases, PT and CD signals are normalized by the two laser powers to compare the two cases. g_CD_ factors are mentioned in the inset. Scale bar = 500 nm.

Inspired by the high PT enhancement at the critical point of CO_2_, we focused on enhancing a MCD signal. We have measured the MCD of a single magnetite nanoparticle cluster of size of about 25 to 35 nm inside a capillary at the critical point of CO_2_ and in hexadecane as shown in Figure 4. These clusters are formed during the sample preparation inside the capillary from single 20 nm magnetite particles. The heating and probe laser powers were 102 mW and 18 mW, respectively, in hexadecane and 15.5 and 1 mW, respectively, in CO_2_. Note that we have used about 6.5 and 18 times lower heating and probe laser powers, respectively, in CO_2_ compared to that in hexadecane. In hexadecane, we observed very weak CD signals of nanoparticles in the absence of magnetic field, probably due to geometric CD. Indeed, random chiral shapes of magnetite particles may lead to weak CD signals because of the weakness or absence of depolarization fields, which are very strong for plasmonic nanoparticles. However, in super-critical CO_2_, the CD signal of nanoparticles in the absence of magnetic field is much stronger than that in hexadecane due to the enhancement of the CD signal in CO_2_. Under an applied magnetic field, a strong MCD signal is observed, which flips sign upon reversal of the magnetic field direction. This is a signature of MCD. The g_CD_ factor of the MCD signal in both cases is between 1.5 and 4.2%. As mentioned above, for MCD measurements in CO_2_, we used much lower heating and probe powers than in hexadecane. By normalizing PT and CD signals with heating and probe laser powers and considering similar cluster sizes in two measurements, an enhancement factor of about 100 is obtained for measurements in CO_2_ as can be seen in Figure 4. The measurement was performed at 37 °C and 7.5 MPa which is close to the critical point but not at the critical point. We want to stress here that the enhancement factor we find for magnetite particles is a qualitative estimation.

## Conclusions

In summary, we have demonstrated the enhancement of the PT signal of a single 30 nm AuNP in CO_2_ by more than 1000-fold, similarly to the enhancement obtained in xenon and reported previously by our group.^21^ The advantages of using CO_2_ and a capillary-based sample preparation are that (i) CO_2_ is much cheaper than xenon, (ii) the critical temperature of CO_2_ being higher than the room temperature, the sample should be heated rather than cooled, and (iii) the capillary facilitates the sample preparation as compared to our previous design of the pressure cell. However, the inhomogeneous distribution of PT signals in CO_2_ needs to be carefully interpreted. In addition, we have demonstrated the enhancement of the MCD signal of a single magnetite nanoparticle cluster. Our measurements in CO_2_ pave the way to studies of many other nanomaterials which cannot withstand high excitation powers. Near-critical PT microscopy can then be applied to a number of systems. Single molecules of conjugated polymers suffer photodamage under strong illumination. Similarly, semiconductor quantum dots and perovskite nanocrystals present complex photophysics and multi-exciton transitions and should be investigated at as low an excitation intensity as possible. Many biological structures as well as plasmonic structures such as gold nanorods are prone to denaturation or reshaping under heavy illumination. Finally, the dynamics of superparamagnetic nanoparticles is very sensitive to temperature. Reduced excitation intensities will enable studies of these systems at temperatures closer to ambient.

## Supporting Information Available

Simulation of PT enhancement factor in xenon; enhancement factor for the figure of merit in CO_2_ and xenon; simulation of the CD signal of a single chiral nanoparticle; PSF of PT and MCD signals of a single particle inside a capillary; PT enhancement factor map in xenon; and schematic of the whole sample holder (PDF)

## ABBREVIATIONS

PT: photothermal
CD: circular dichroism
PT CD: photothermal circular dichroism
PT MCD: photothermal magnetic circular dichroism.
