# Supplementary material for: Thousand-Fold Enhancement of Photothermal Signals in Near-Critical CO2
Source: J Phys Chem C Nanomater Interfaces. 2023 Feb 9;127(7):3619–25. doi: 10.1021/acs.jpcc.2c08575 (PMC9969513; doi:10.1021/acs.jpcc.2c08575)
Supplement: Supplementary file 1 — jp2c08575_si_001.pdf [file jp2c08575_si_001.pdf]

Supporting Information for

Thousand-Fold Enhancement of Photothermal Signals in Near-Critical CO_2

Yonghui Wang[1,2,‡], Subhasis Adhikari[1,‡], Harmen van der Meer[1], Junyan Liu[2], Michel Orrit[1,]*

‡ These authors contributed equally to this work

1 Huygens-Kamerlingh Onnes Laboratory, Leiden University; 2300 RA Leiden, The Netherlands

2 School of Mechatronics Engineering, Harbin Institute of Technology; Harbin 150001, P. R. China

*Corresponding authors: orrit@physics.leidenuniv.nl; ljywlj@hit.edu.cn

Simulation of photothermal enhancement factor in xenon:

We have calculated the expected photothermal (PT) enhancement factor (with respect to the photothermal signal in hexadecane) in xenon at various temperatures and pressures near the critical point using a COMSOL simulation described in the main text. Figure S1 shows the PT enhancement factor map. The maximum enhancement expected at the critical point of xenon is about 4,200 which is about 2.8 times larger than that obtained in CO_2. Below the critical point, near the three data points (i.e., 16°C, 5.7 MPa; 15°C, 5.6 MPa and 14°C, 5.5 MPa), the

photothermal signal varies discontinuously due to the liquid-gas phase transition.

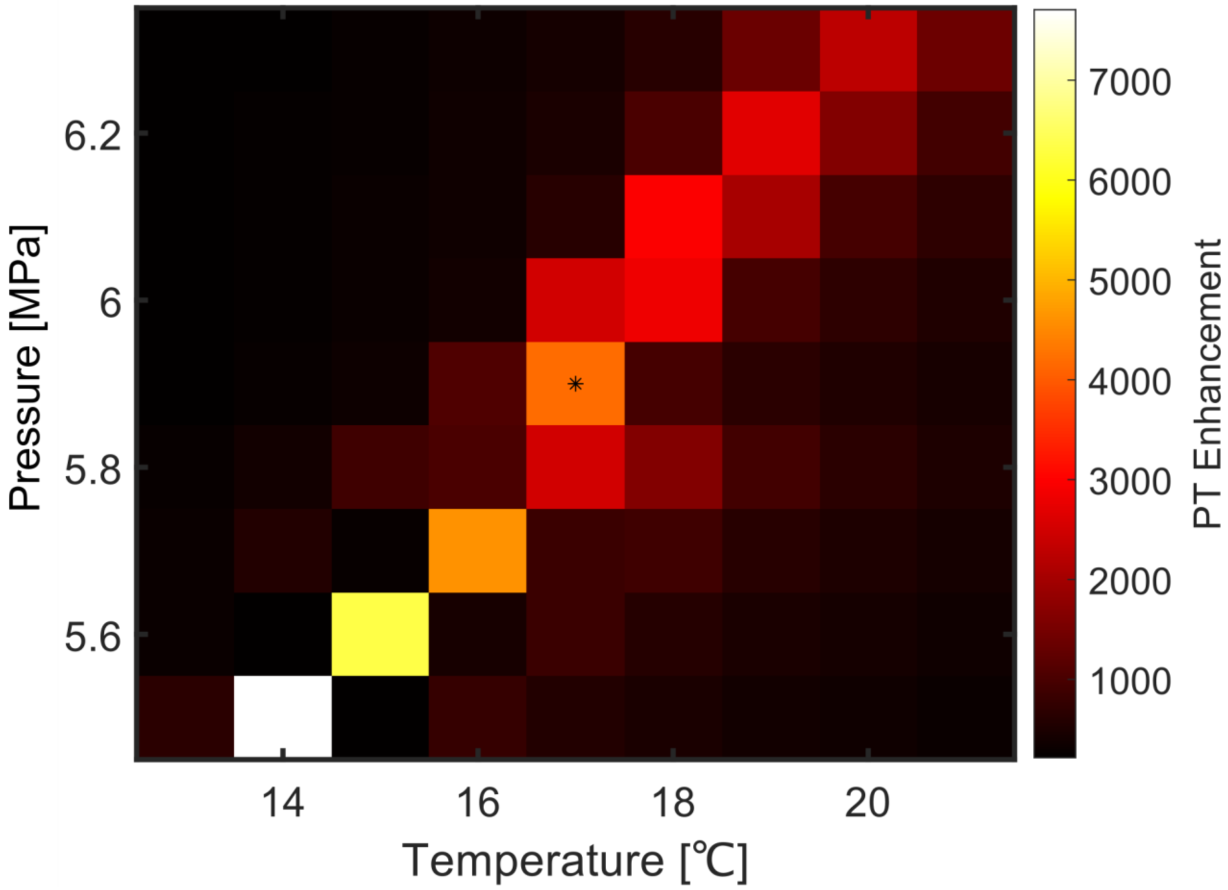

Figure S1: Simulated PT enhancement factor map in xenon at various temperatures and pressures near the critical point (marked with star sign) using a COMSOL simulation described in the main text.

<u>Enhancement factor for figure of merit in CO_2 and xenon</u>:

To compare the photothermal signal qualitatively in two different media, ref.[1] proposed to calculate the figure of merit (FOM) and strength of the photothermal medium. FOM is $n \times dn/dT \times (1/k)$ and the photothermal strength is $n \times dn/dT \times (1/C_p)$ where n is the refractive index, dn/dT is the thermo-refractive coefficient, k is the thermal conductivity and C_p is the heat

capacity of the medium. We have calculated FOM and photothermal strength in both CO_2 and xenon and calculated the enhancement factor map of these quantities with respect to hexadecane as shown in Figure S2 and S3, respectively.

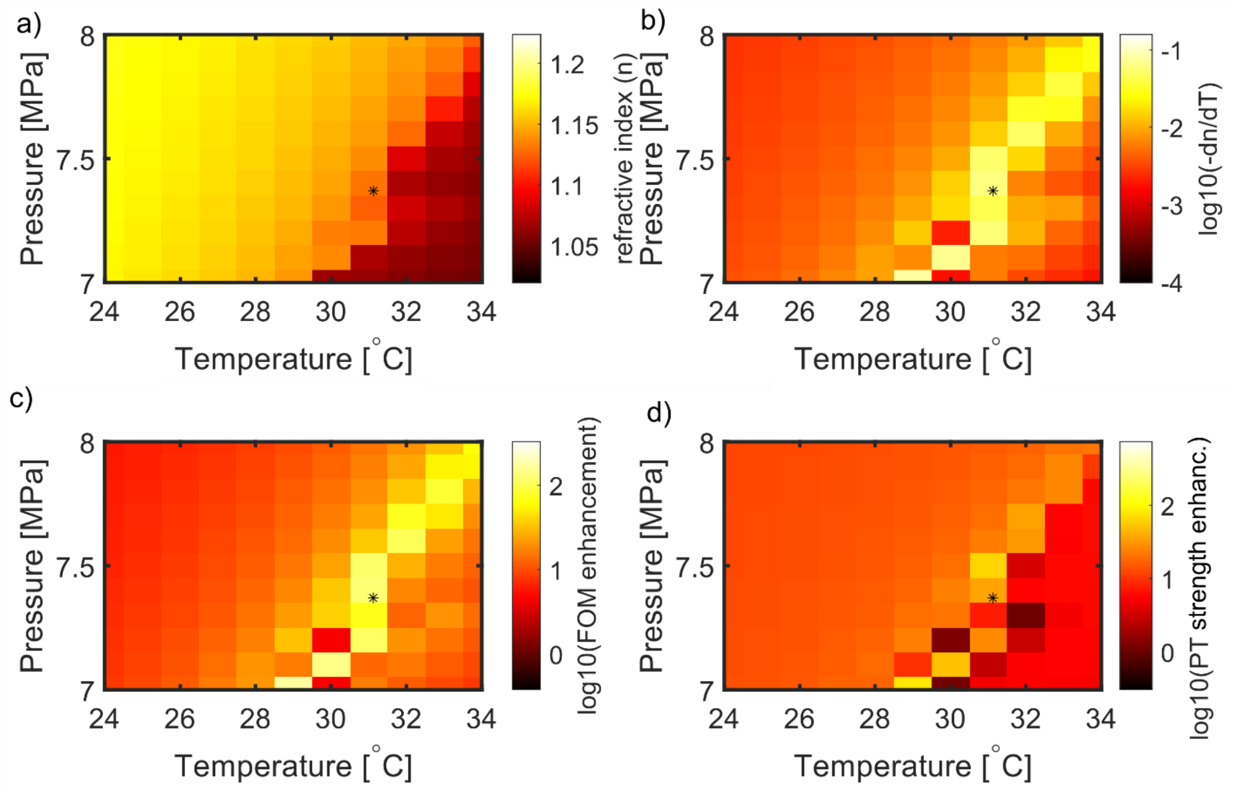

Figure S2: (a) refractive index (n), (b) thermo-refractive coefficient (dn/dT), (c) figure of merit (FOM) enhancement factor and (d) photothermal strength enhancement factor calculated in CO_2 at different temperatures and pressures near the critical point as marked by the star sign. The enhancement factor is calculated with respect to hexadecane. Refractive index, dn/dT, thermal conductivity and heat capacity data are taken from NIST Chemistry WebBook.

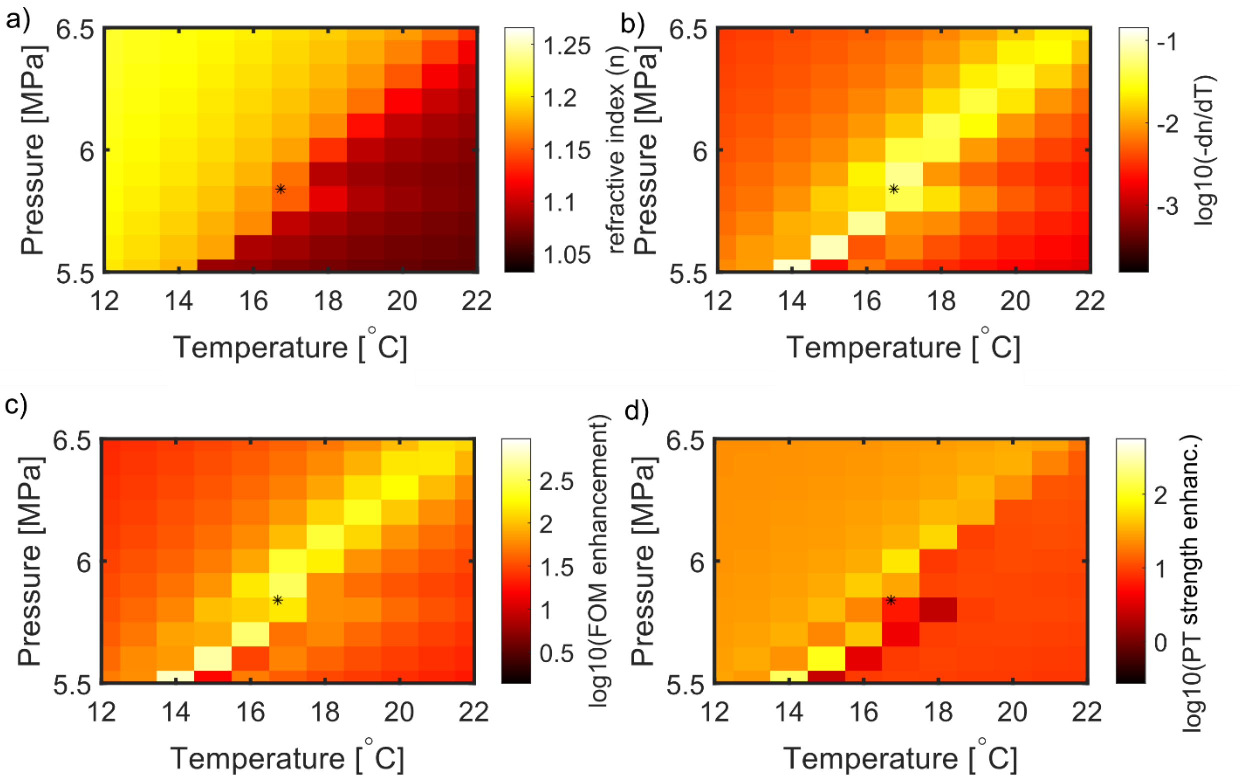

Figure S3: (a) refractive index (n), (b) thermo-refractive coefficient (dn/dT), (c) figure of merit (FOM) enhancement factor and (d) photothermal strength enhancement factor calculated in xenon at different temperatures and pressures near the critical point as marked by the star sign. The enhancement factor is calculated with respect to hexadecane. Refractive index, dn/dT, thermal conductivity and heat capacity data are taken from NIST Chemistry WebBook.

<u>Simulation of the CD signal of a single chiral nanoparticle</u>

Figure S4 shows a CD enhancement factor map of a single chiral nanoparticle expected using the COMSOL simulation as described in the main text. In the simulation, the absorption cross section and g_{CD} factor of the particle are taken as 2×10^{-12} cm^2 and 0.02, respectively. The heating and probe laser powers are taken as 70 μW and 10 μW, respectively. Similar to photothermal, the maximum CD enhancement factor expected at the critical point is about 1,500. The g_{CD} factor is

also calculated from the simulation as the CD signal normalized by photothermal signal. A map of g_{CD} factor obtained from the simulation at different temperatures and pressures is shown in Figure S4. These results show that the CD signal can be enhanced by more than a thousand-fold, as the photothermal signal. As discussed in the main text, in our measurements of the MCD signal of single magnetite nanoparticle clusters, we have obtained a qualitative estimation of the enhancement factor of about 100, which is an order of magnitude lower than the maximum enhancement factor predicted by the simulation. The main reason is that in the experiment, because we had to use relatively high laser powers, the surface temperature rise at the particle brought the system far from the critical point, and the enhancement was not optimal.

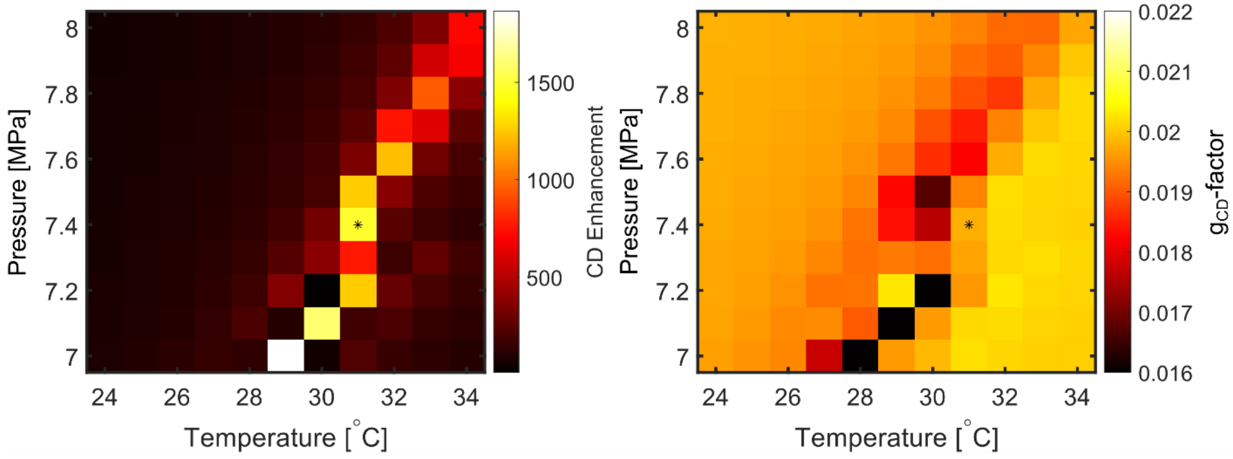

Figure S4: (Left) Expected CD enhancement factor map of a single chiral nanoparticle with g_{CD} factor of 0.02 in CO_2 at different temperatures and pressures. (Right) The calculated g_{CD} factor map obtained from the COMSOL simulation.

<u>PSF of PT and MCD signal of a single particle inside a capillary:</u>

Photothermal and MCD images of a single magnetite nanoparticle in hexadecane are shown in Figure S5. The corresponding line profiles are shown in Figure S6. The lateral and longitudinal

sizes for the point-spread functions (PSF's) in PT and MCD are mentioned in the inset. Both lateral and longitudinal sizes are larger than the PSF's dimensions in the standard confocal geometry. The best diffraction-limited lateral and axial resolutions with the objective are $\lambda/(2\times NA)$ and $(2\times n\times\lambda)/NA^2$ where λ is the wavelength, n is the refractive index of the medium and NA is the numerical aperture. In our case, λ= 780 nm, n= 1.51 and NA= 1.45. Therefore, the expected best lateral and axial resolutions are 269 nm and 1.12 µm. In our experiment, the lateral and axial resolutions are 750 nm and 3.1 µm for photothermal, and 550 nm and 3.2 µm for CD measurements. With an effective numerical aperture NA of 0.8, the diffraction-limited lateral and axial resolutions would be about 488 nm and 3.6 µm which are close to the experimental observations. This reduction of numerical aperture is probably due at least partly to vignetting of the beam by the capillary's useful width, as discussed in the main text.

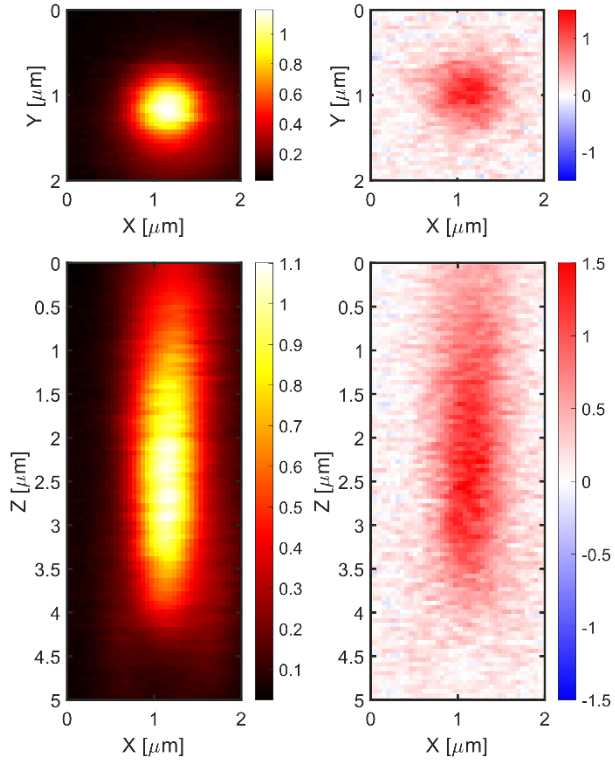

Figure S5: (Left) photothermal images in (top) XY and (bottom) XZ planes. (Right) MCD images in (top) XY and (bottom) XZ planes. The MCD signal was from a single magnetite nanoparticle cluster of about 30 nm at B=+ 280 mT.

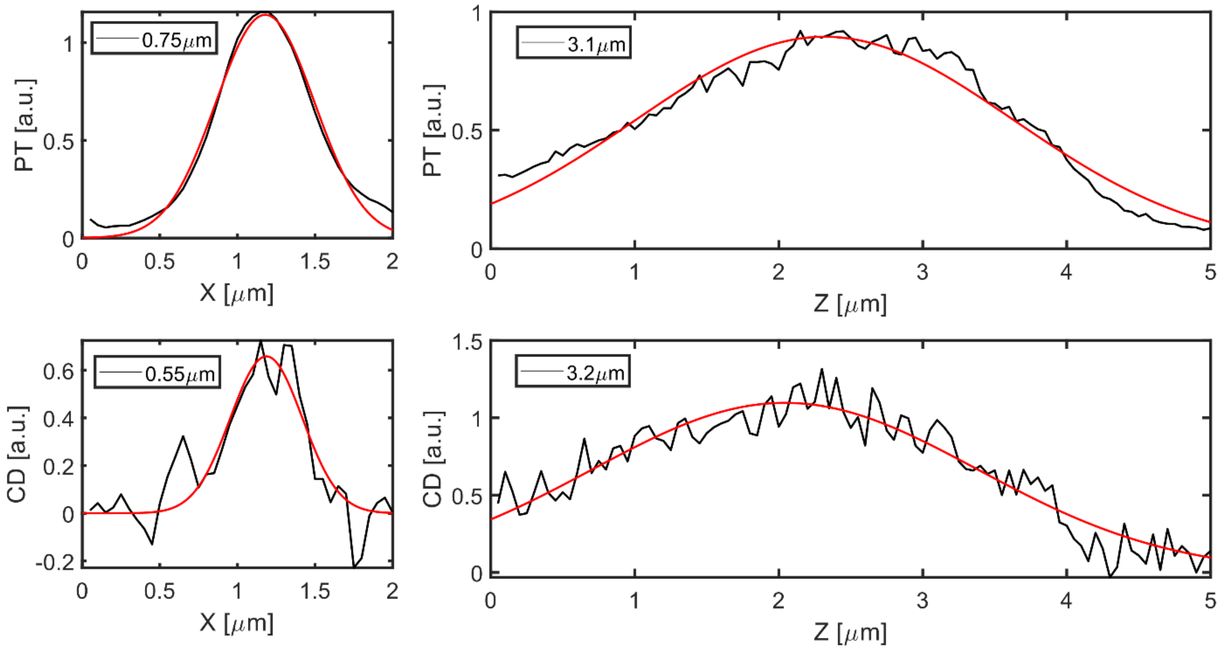

Figure S6: The line profiles correspond to the PT and MCD images shown in Figure S5, accordingly. The full width at half maximum (FWHM) is mentioned in the inset.

<u>Photothermal enhancement factor map in xenon:</u>

Figure S7 shows an enhancement factor map for single 30 nm gold nanoparticles measured inside a capillary in xenon. The maximum enhancement factor is found to be about 2,200, which is quite similar to the value obtained in CO_2, although a factor of 2.5 lower than expected from the simulation. The mismatch between simulation and experiment may be assigned to the low purity of our xenon gas.

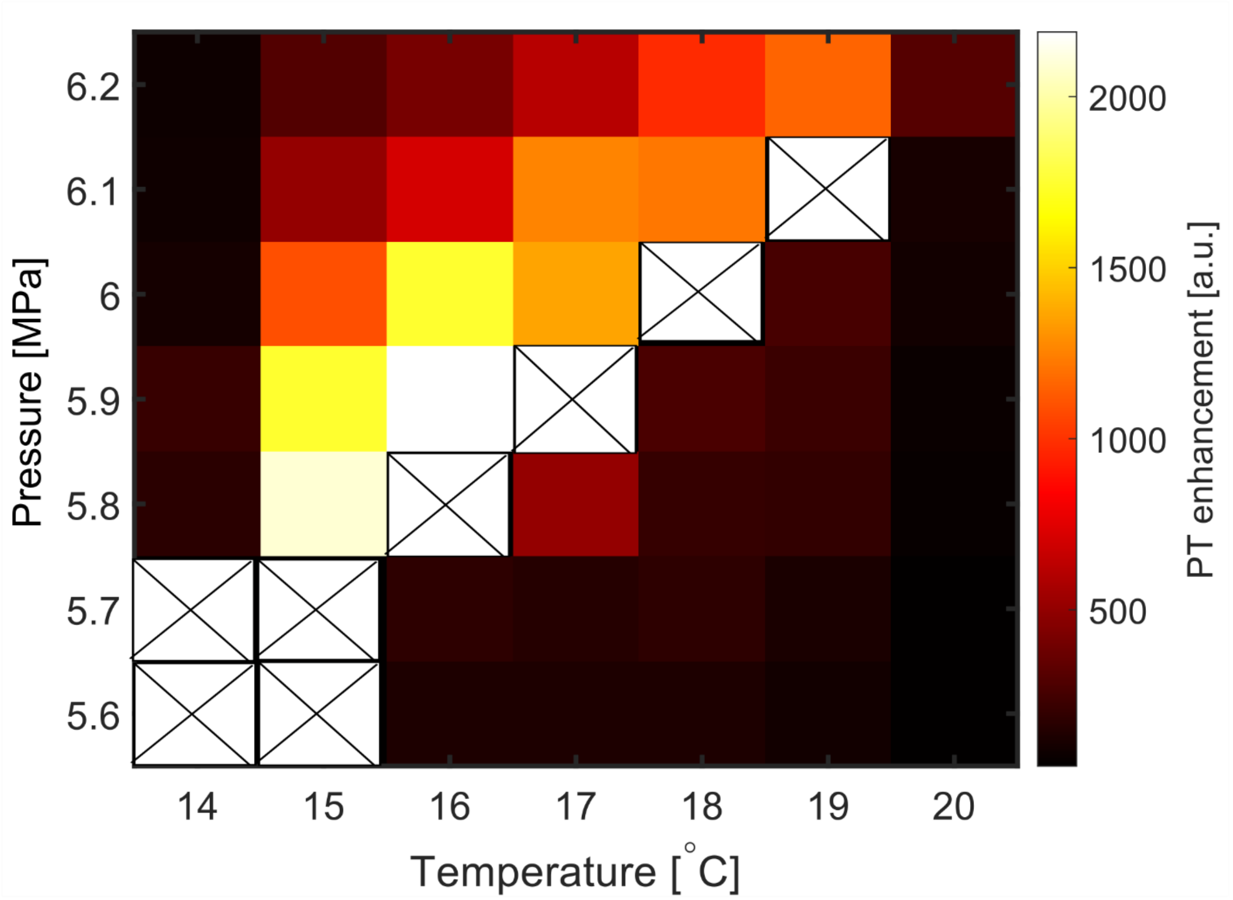

Figure S7: Photothermal enhancement factor map measured at various temperatures and pressures near the critical point of xenon (16°C and 5.9 MPa). The cross symbols indicate large fluctuations of the PT signal. We therefore could not calculate any enhancement factor at these temperatures and pressures. The large fluctuations at temperatures and pressures below the critical point are due to the liquid-gas phase transition and above the critical point are probably due to high dn/dT values and high sensitivity of photothermal signals to a small perturbation.

Schematic of the whole sample holder

A schematic of the whole sample holder containing capillary pressure cell, heating elements and temperature sensor is shown in Figure S8.

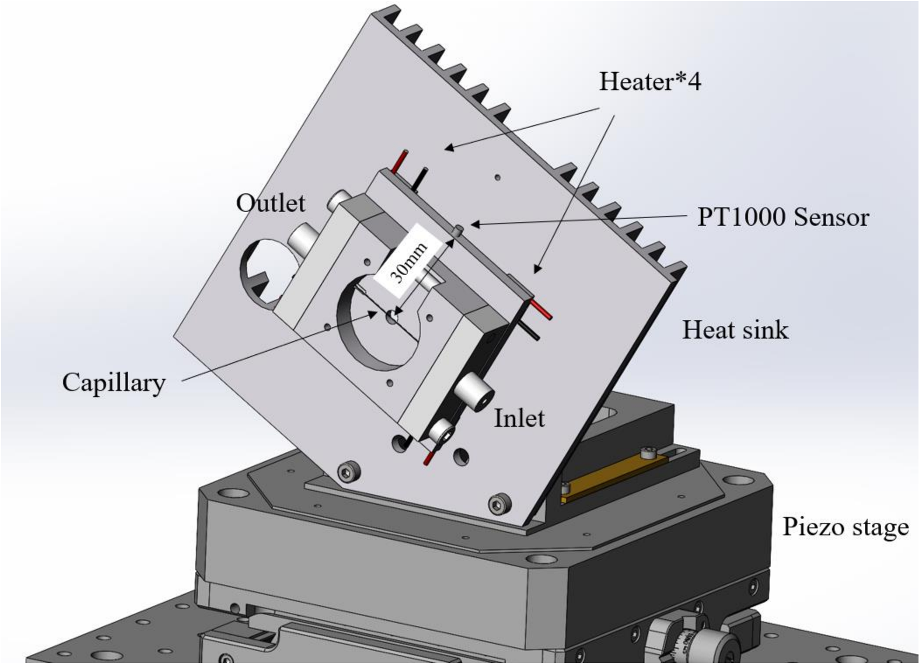

Figure S8: The schematic of the whole sample holder. The temperature probe is a PT1000 sensor which is 30 mm away from the focus of the objective. The distance causes a significant temperature difference between the sample temperature and that of the probe.

References

(1) Gaiduk, A.; Ruijgrok, P. V.; Yorulmaz, M.; Orrit, M. Detection Limits in Photothermal Microscopy. *Chem. Sci.* **2010**, *1* (3), 343–350. https://doi.org/10.1039/C0SC00210K.
